# *N*-(6-Meth­oxy­pyridin-2-yl)-1-(pyridin-2-ylmeth­yl)-1*H*-pyrazole-3-carboxamide: crystal structure and Hirshfeld surface analysis

**DOI:** 10.1107/S2056989018011477

**Published:** 2018-08-16

**Authors:** Vivek C. Ramani, Rina D. Shah, Mukesh M. Jotani, Edward R. T. Tiekink

**Affiliations:** a Department of Chemistry, M. G. Science Institute, Navrangpura, Ahmedabad, Gujarat 38009, India; b Department of Physics, Bhavan’s Sheth R. A. College of Science, Ahmedabad, Gujarat 380 001, India; c Research Centre for Crystalline Materials, School of Science and Technology, Sunway University, 47500 Bandar Sunway, Selangor Darul Ehsan, Malaysia

**Keywords:** crystal structure, pyrazol­yl, pyrid­yl, Hirshfeld surface analysis

## Abstract

The title compound, C_16_H_15_N_5_O_2_, adopts the shape of the letter *L* with the dihedral angle between the outer pyridyl rings being 78.37 (5)°; the dihedral angles between the central pyrazolyl ring (r.m.s. deviation = 0.0023 Å) and the methyl­ene-bound pyridyl and methyoxypyridyl rings are 77.68 (5) and 7.84 (10)°, respectively. Intra­molecular amide-N—H⋯N(pyrazol­yl) and pyridyl-C—H⋯O(amide) inter­actions are evident and these preclude the participation of the amide-N—H and O atoms in inter­molecular inter­actions. The most notable feature of the mol­ecular packing is the formation of linear supra­molecular chains aligned along the *b*-axis direction mediated by weak carbonyl-C=O⋯π(triazol­yl) inter­actions. An analysis of the calculated Hirshfeld surfaces point to the importance of H⋯H (46.4%), C⋯H (22.4%), O⋯H (11.9%) and N⋯H (11.1%) contacts in the crystal.

## Abstract 2

The title mol­ecule adopts the shape of the letter *L* as the dihedral angle between the pyridyl rings is 78.37 (5)°. Linear supra­molecular chains are found in the crystal mediated by weak carbonyl-C=O⋯π(triazol­yl) inter­actions.

## Chemical context   

Amide bond formation involving acid–amine coupling is an important synthetic tool for the manufacture of pharmaceuticals and fine chemicals (Schuele *et al.*, 2008 ▸). The use of a variety of acid–amine coupling agents, most commonly carbodi­imides and onium salts such as phospho­nium as well as ammonium salts, for amide bond synthesis has been reviewed (Al-Warhi *et al.* 2012 ▸; Urich *et al.*, 2014 ▸). In this context, *n*-propane­phospho­nic acid anhydride (T3P) has proved to be an excellent reagent for amide or peptide bond formation. The synthesis of amide bonds utilizing T3P offers high yields, low epimerization and avoids the use of haza­rdous additives such as explosive hy­droxy­benzotriazole (HOBt). Further, reactions occur with high yields and lead to the easy removal of the by-products with a simple work-up, overall resulting in the formation of high-quality product. In addition, it is noted that the T3P reagent is non-toxic and non-allergenic (Joullie & Lassen, 2010 ▸; Fennie & Roth, 2016 ▸). Moreover, amine bond formation between pyrazole and pyrimidine ring systems can lead to the formation of biologically accepted ingredients such as AM251 (Xi *et al.*, 2006 ▸), as a CB1 cannabinoid receptor antagonist, and Meclinertant (SR48692; Liu *et al.*, 2017 ▸), a neurotensinreceptor (NTS) antagonist. The combination of such moieties can also lead to mol­ecules with anti-tuberculosis, anti-cancer, anti-bacterial and anti-fungal activities (Fustero *et al.*, 2009 ▸; Pal *et al.*, 2012 ▸; Dar & Shamsuzzaman, 2015 ▸; Sapra *et al.*, 2016 ▸). As part of our studies in this area, acid–amine coupling between heterocycles such as pyrazole and pyridine using efficient coupling reagents such as T3P was performed; herein, the crystal and mol­ecular structures of (I) are described along with an analysis of its calculated Hirshfeld surface.
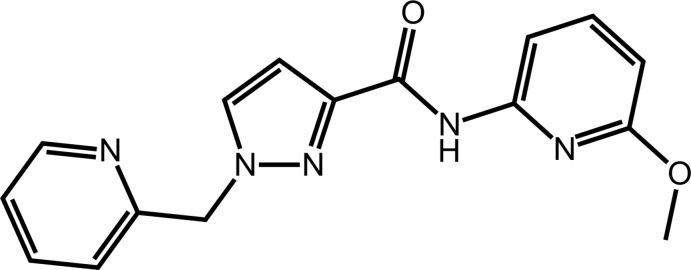


## Structural commentary   

**Figure 1:**
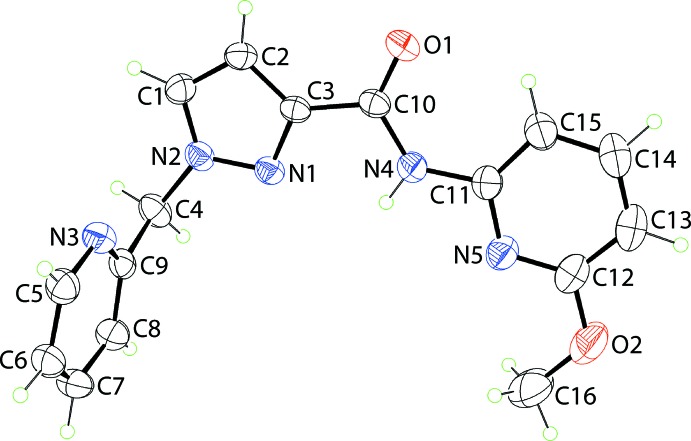
The mol­ecular structure of (I), showing displacement ellipsoids at the 50% probability level.

The mol­ecular structure of (I), Fig. 1 ▸, comprises an almost planar bi-substituted pyrazolyl ring with the r.m.s. deviation of the fitted atoms being 0.0023 Å. Connected to the ring at the N2 position is a methyl-2-pyridyl residue with the dihedral angle between the five- and six-membered rings being 77.68 (5)°, indicating an almost orthogonal relationship. A substituted amide (C10/N4/O1) group is connected at the C3-position, which is approximately co-planar with the pyrazolyl ring, forming a dihedral angle of 3.5 (3)°. The dihedral angle between the amide atoms and the appended N5-pyridyl ring is 4.4 (3)°, indicating a co-planar relationship. The dihedral angle between the pyridyl rings in (I) of 78.37 (5)° indicates that the mol­ecule has an approximate *L*-shape. The amide-N4—H4*N* atom is flanked on either side by the pyrazolyl-N1 and pyridyl-N5 atoms and in the same way, the amide-O1 atom accepts a weak intra­molecular inter­action from the C15—H15 grouping; see Table 1 ▸ for geometric data characterizing these inter­actions. Finally, the meth­oxy group is approximately co-planar with the pyridyl ring to which it is attached, as seen in the C16—O2—C12—N5 torsion angle of 4.2 (3)°.

## Supra­molecular features   

**Figure 2:**
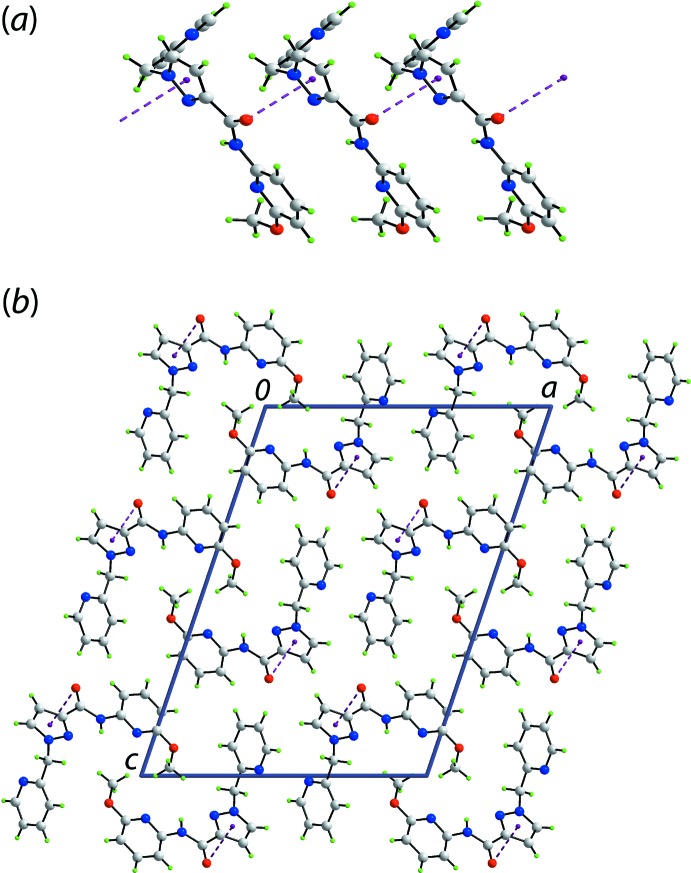
Supra­molecular association in the crystal of (I): (*a*) a view of the supra­molecular chain along the *b*-axis direction sustained by carbonyl-C—O⋯π(triazol­yl) inter­actions shown as purple dashed lines and (*b*) a view of the unit-cell contents shown in projection down the *b* axis.

The mol­ecular packing of (I) is largely devoid of structure-directing inter­actions as the key amide atoms are involved in intra­molecular contacts. The only identified directional inter­action less than van der Waals separations (Spek, 2009 ▸) is a carbonyl-C10=O2⋯π(triazol­yl) contact, Table 1 ▸. As illus­trated in Fig. 2 ▸ *a*, these lead to linear supra­molecular chains aligned along the *b*-axis direction. The supra­molecular chains pack without specific inter­actions between them, Fig. 2 ▸ *b*.

## Hirshfeld surface analysis   

**Figure 3:**
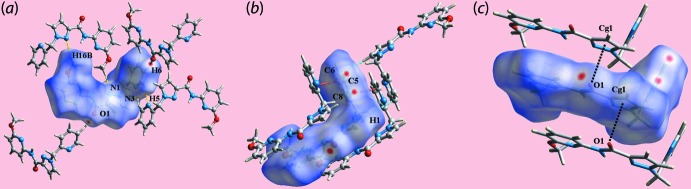
Three views of the Hirshfeld surface for (I) mapped over *d*_norm_ in the range −0.093 to +1.418 a.u. highlighting (*a*) short inter­atomic O⋯H/H⋯O (yellow dashed lines) and N⋯H/H⋯N (black) contacts dashed lines, (*b*) C⋯C (red) and C⋯H/H⋯C (sky-blue) contacts and (*c*) C=O⋯π contacts (black dotted lines).

**Figure 4:**
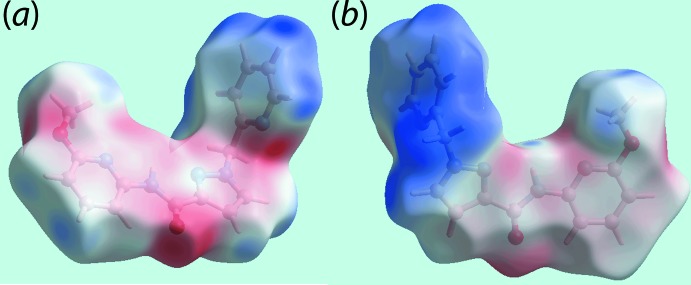
Two views of the Hirshfeld surface mapped over the electrostatic potential in the range −0.080 to +0.044 a.u. The red and blue regions represent negative and positive electrostatic potentials, respectively.

The Hirshfeld surfaces calculated for (I) were performed in accord with recent studies (Jotani *et al.*, 2016 ▸) and provide additional information on the influence of short inter­atomic contacts influential in the mol­ecular packing. On the Hirshfeld surfaces mapped over *d*_norm_ in Fig. 3 ▸, the presence of diminutive red spots near the pyrazole-N1, methyl-H16*B*, pyridyl-N3 and pyridyl-H5 atoms are indicative of short inter­atomic N⋯H/H⋯N contacts (Table 2 ▸). In addition, the presence of diminutive red spots near the carbonyl-O1 and pyridyl-H6 atoms on the surface connect the mol­ecules through short inter­atomic O⋯H/H⋯O contacts (Table 2 ▸) are highlighted through black dashed lines in Fig. 3 ▸ *a*. The faint-red spots appearing near the pyridyl-C5, C6 and C8 atoms and the pyrazolyl-H1 atom in Fig. 3 ▸ *b* represent the short inter­atomic C⋯C and C⋯H/H⋯C contacts (Table 3 ▸) between these atoms. The inter­molecular C=O⋯π contacts connecting the mol­ecules along the *b*-axis direction are illustrated in Fig. 3 ▸ *c*. The weak inter­molecular inter­actions described above are also viewed as the blue and red regions near the respective atoms on the Hirshfeld surfaces mapped over the calculated electrostatic potential shown in Fig. 4 ▸.

**Figure 5:**
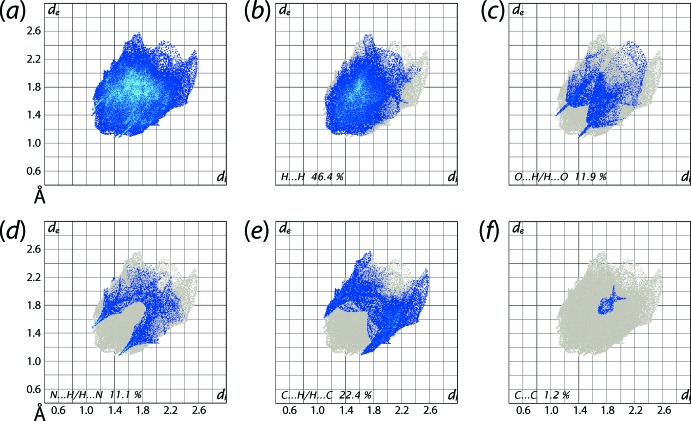
(*a*) The full two-dimensional fingerprint plot for (I) and (*b*)-(*f*) those delineated into H⋯H, O⋯H/H⋯O, N⋯H/H⋯N, C⋯H/H⋯C and C⋯C, contacts, respectively.

The overall two-dimensional fingerprint plot for (I), Fig. 5 ▸ *a*, and those delineated into H⋯H, N⋯H/H⋯N, O⋯H/H⋯O, C⋯H/H⋯C and C⋯C contacts (McKinnon *et al.*, 2007 ▸) are illustrated in Fig. 5 ▸ *b*–*f*, respectively, and the percentage contributions from the different inter­atomic contacts to the Hirshfeld surface are summarized in Table 3 ▸. The greatest, *i.e*. 46.4%, contribution to the Hirshfeld surfaces are from H⋯H contacts and indicates the significance of dispersive forces on the mol­ecular packing as the inter­atomic distances involving these contacts are greater than the sum of van der Waals radii. The short inter­atomic O⋯H/H⋯O and C⋯H/H⋯C contacts in the crystal structure of (I) are characterized as the pair of thin needle-like and forceps-like tips at *d*_e_ + *d*_i_ ∼ 2.5 Å and 2.7 Å, respectively, in the corresponding delineated fingerprint plots Fig. 5 ▸ *c* and *e*. The pair of spikes with the tips at *d*_e_ + *d*_i_ ∼ 2.6 Å and the regions of green points aligned in the fingerprint plot delineated into N⋯H/H⋯N contacts, Fig. 5 ▸ *d*, are indicative of short N⋯H inter­atomic contacts (Table 2 ▸). In the fingerprint plot delineated into C⋯C contacts, Fig. 5 ▸ *f*, the presence of points at *d*_e_ + *d*_i_ < 3.4 Å, *i.e*. less than the sum of van der Waals radii, are due to short inter­atomic C5⋯C8 contacts involving pyridyl-carbon atoms (Fig. 3 ▸ *b*) although the contribution from these contacts is relatively small. The notable percentage contributions from O⋯N/N⋯O and C⋯O/O⋯C contacts to the Hirshfeld surfaces (Table 2 ▸) in the crystal arise from the presence of the inter­molecular C=O⋯π contacts. The inter­atomic N⋯N contacts show no significant contribution to the packing of (I).

## Database survey   

The 1,3 N—C and C—C(=O)N(H)—C substitution pattern observed in (I), with hydrogen atoms at the C1 and C2 positions, is unprecedented in structural chemistry according to a search of the Cambridge Structural Database (CSD Version 5.39, May update; Groom *et al.*, 2016 ▸). There are considerably more examples of structures with substituents at one of and at both the C1 and C2 positions but none of these are substituents are pyridyl groups.

## Synthesis and crystallization   

1*H*-Pyrazole-4-carb­oxy­lic acid (0.0446 mol) was treated with diiso­propyl­ethyl amine (0.0669 mol) and 1-propane phospho­nic acid (T3P) (0.0669 mol) in dimethyl formamide (10 ml) at 273 K for 15 min. Then, 6-meth­oxy­pyridin-2-amine (0.0490 mol) was added at 273 K. The reaction mixture was heated at 353 K for 3 h. After completion of the reaction, the product was extracted with ethyl acetate and the excess solvent was removed under vacuum. The product was recrystallized using methanol as solvent to yield 1-(6-meth­oxy­pyridin-2-ylmeth­yl)-1*H*-pyrazole-4-carb­oxy­lic acid. This product (0.0246 mol) and 2-(chloro­meth­yl)pyridine (0.0295 mol) were dissolved in acetone (10 ml), potassium carbonate (0.0369) was added and the reaction mixture was heated at 329 K for 5 h. After completion of the reaction, the product was extracted with ethyl acetate twice (5 ml) and the extract was concentrated under vacuum. The product was washed with diethyl ether (3 ml) and recrystallized from methanol solution to obtain the title compound, (I), as colourless blocks in 88% yield. M.p. 414-415 K. CHN analysis: calculated. C, 62.13; H, 4.89; N, 22.64%; observed: C, 62.06; H, 4.81; N, 22.84%.

## Refinement details   

Crystal data, data collection and structure refinement details are summarized in Table 4 ▸. The carbon-bound H atoms were placed in calculated positions (C—H = 0.93–0.97 Å) and were included in the refinement in the riding-model approximation, with *U*_iso_(H) set to 1.2–1.5*U*_eq_(C). The N-bound H atoms was refined with a distance restraint of 0.86±0.01 Å, and with *U*_iso_(H) = 1.2*U*_eq_(N).

## Supplementary Material

Crystal structure: contains datablock(s) I, global. DOI: 10.1107/S2056989018011477/hb7767sup1.cif

Structure factors: contains datablock(s) I. DOI: 10.1107/S2056989018011477/hb7767Isup2.hkl

Supporting information file. DOI: 10.1107/S2056989018011477/hb7767Isup3.cml

CCDC reference: 1861657

Additional supporting information:  crystallographic information; 3D view; checkCIF report

## Figures and Tables

**Table 1:** Intra- and intermolecular interactions (Å, °) for (I) *Cg*1 is the centroid of the N1/N2/C1–C3 ring.

*D*—H⋯*A*	*D*—H	H⋯*A*	*D*⋯*A*	*D*—H⋯*A*
N4—H4*N*⋯N1	0.86 (1)	2.24 (2)	2.6939 (18)	113 (1)
C15—H15⋯O1	0.93	2.33	2.909 (2)	120
C10—O1⋯*Cg*1^i^	1.22 (1)	3.42 (1)	3.5486 (16)	86 (1)

Symmetry code: (i) 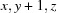.

**Table 2:** Summary of short inter­atomic contacts (Å) in (I)

Contact	Distance	Symmetry operation
O1⋯H6	2.47	*x*, 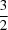 − *y*, 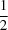 + *z*
N1⋯H16*B*	2.58	−*x*, 1 − *y*, −*z*
N3⋯H5	2.54	1 − *x*, 1 − *y*, −*z*
C6⋯H1	2.72	1 − *x*, − *y*, *z*
C5⋯C8	3.380 (3)	*x*, 1 + *y*, *z*

**Table 3:** Percentage contributions of inter­atomic contacts to the Hirshfeld surface for (I)

Contact	Percentage contribution
H⋯H	46.4
O⋯H/H⋯O	11.9
N⋯H/H⋯N	11.1
C⋯H/H⋯C	22.4
C⋯N/N⋯C	3.5
C⋯O/O⋯C	1.9
N⋯N	1.3
C⋯C	1.2
N⋯O/O⋯N	0.4

**Table 4:** Experimental details

Crystal data	
Chemical formula	C_16_H_15_N_5_O_2_
*M*_r_	309.33
Crystal system, space group	Monoclinic, *P*2_1_/*c*
Temperature (K)	293
*a*, *b*, *c* (Å)	15.8867 (15), 4.6473 (4), 21.6740 (19)
β (°)	108.623 (3)
*V* (Å^3^)	1516.4 (2)
*Z*	4
Radiation type	Mo *K*α
μ (mm^−1^)	0.09
Crystal size (mm)	0.47 × 0.43 × 0.28

Data collection	
Diffractometer	Rigaku SCX mini
Absorption correction	Multi-scan (*REQAB*; Rigaku, 1998 ▸)
*T*_min_, *T*_max_	0.808, 0.974
No. of measured, independent and observed [*I* > 2σ(*I*)] reflections	14205, 3458, 2354
*R*_int_	0.031
(sin θ/λ)_max_ (Å^−1^)	0.649

Refinement	
*R*[*F* ^2^ > 2σ(*F* ^2^)], *wR*(*F* ^2^), *S*	0.042, 0.117, 1.02
No. of reflections	3458
No. of parameters	212
No. of restraints	1
H-atom treatment	H atoms treated by a mixture of independent and constrained refinement
Δρ_max_, Δρ_min_ (e Å^−3^)	0.13, −0.17

Computer programs: *CrystalClear-SM Expert* (Rigaku, 2011 ▸), *SHELXS97* (Sheldrick, 2008 ▸), *SHELXL2014* (Sheldrick, 2015 ▸), *ORTEP-3 for Windows* (Farrugia, 2012 ▸), *DIAMOND* (Brandenburg, 2006 ▸) and *publCIF* (Westrip, 2010 ▸).

## supplementary crystallographic information

### Crystal data

**Table:**

C_16_H_15_N_5_O_2_	*F*(000) = 648
*M**_r_* = 309.33	*D*_x_ = 1.355 Mg m^−^^3^
Monoclinic, *P*2_1_/*c*	Mo *K*α radiation, λ = 0.71073 Å
*a* = 15.8867 (15) Å	Cell parameters from 11059 reflections
*b* = 4.6473 (4) Å	θ = 3.2–27.7°
*c* = 21.6740 (19) Å	µ = 0.09 mm^−^^1^
β = 108.623 (3)°	*T* = 293 K
*V* = 1516.4 (2) Å^3^	Block, colourless
*Z* = 4	0.47 × 0.43 × 0.28 mm

### Data collection

**Table:**

Rigaku SCX mini diffractometer	2354 reflections with *I* > 2σ(*I*)
Detector resolution: 6.849 pixels mm^-1^	*R*_int_ = 0.031
ω scans	θ_max_ = 27.5°, θ_min_ = 3.8°
Absorption correction: multi-scan (*REQAB*; Rigaku, 1998)	*h* = −19→20
*T*_min_ = 0.808, *T*_max_ = 0.974	*k* = −6→6
14205 measured reflections	*l* = −28→28
3458 independent reflections	

### Refinement

**Table:**

Refinement on *F*^2^	1 restraint
Least-squares matrix: full	Hydrogen site location: mixed
*R*[*F*^2^ > 2σ(*F*^2^)] = 0.042	H atoms treated by a mixture of independent and constrained refinement
*wR*(*F*^2^) = 0.117	*w* = 1/[σ^2^(*F*_o_^2^) + (0.0534*P*)^2^ + 0.1998*P*] where *P* = (*F*_o_^2^ + 2*F*_c_^2^)/3
*S* = 1.02	(Δ/σ)_max_ < 0.001
3458 reflections	Δρ_max_ = 0.13 e Å^−^^3^
212 parameters	Δρ_min_ = −0.17 e Å^−^^3^

### Special details

**Table:**

Geometry. All esds (except the esd in the dihedral angle between two l.s. planes) are estimated using the full covariance matrix. The cell esds are taken into account individually in the estimation of esds in distances, angles and torsion angles; correlations between esds in cell parameters are only used when they are defined by crystal symmetry. An approximate (isotropic) treatment of cell esds is used for estimating esds involving l.s. planes.

### Fractional atomic coordinates and isotropic or equivalent isotropic displacement parameters (Å^2^)

**Table:**

	*x*	*y*	*z*	*U*_iso_*/*U*_eq_	
O1	0.33267 (7)	0.7478 (2)	0.23734 (5)	0.0605 (3)	
O2	−0.08095 (9)	0.9148 (4)	0.07466 (8)	0.1027 (5)	
N1	0.30289 (8)	0.2514 (2)	0.10788 (5)	0.0434 (3)	
N2	0.36665 (8)	0.0898 (2)	0.09706 (5)	0.0457 (3)	
N3	0.40561 (8)	0.2467 (3)	−0.01587 (6)	0.0511 (3)	
N4	0.20890 (8)	0.6321 (3)	0.15313 (6)	0.0516 (3)	
H4N	0.1920 (10)	0.521 (3)	0.1198 (6)	0.062*	
N5	0.06549 (9)	0.7801 (3)	0.11608 (7)	0.0577 (4)	
C1	0.44689 (10)	0.1427 (3)	0.14031 (7)	0.0517 (4)	
H1	0.5001	0.0558	0.1415	0.062*	
C2	0.43609 (10)	0.3459 (3)	0.18194 (7)	0.0483 (4)	
H2	0.4795	0.4266	0.2172	0.058*	
C3	0.34578 (9)	0.4073 (3)	0.16025 (6)	0.0408 (3)	
C4	0.34470 (12)	−0.1001 (3)	0.04122 (7)	0.0548 (4)	
H4A	0.2866	−0.1833	0.0350	0.066*	
H4B	0.3874	−0.2563	0.0502	0.066*	
C5	0.34405 (10)	0.0471 (3)	−0.02091 (6)	0.0438 (3)	
C6	0.40938 (11)	0.3653 (4)	−0.07088 (7)	0.0567 (4)	
H6	0.4516	0.5076	−0.0679	0.068*	
C7	0.35489 (12)	0.2904 (4)	−0.13146 (8)	0.0615 (5)	
H7	0.3609	0.3760	−0.1686	0.074*	
C8	0.29151 (12)	0.0865 (4)	−0.13579 (8)	0.0663 (5)	
H8	0.2530	0.0310	−0.1762	0.080*	
C9	0.28514 (11)	−0.0364 (4)	−0.07958 (7)	0.0573 (4)	
H9	0.2417	−0.1733	−0.0814	0.069*	
C10	0.29654 (9)	0.6120 (3)	0.18811 (6)	0.0442 (3)	
C11	0.14308 (10)	0.8033 (3)	0.16395 (7)	0.0488 (4)	
C12	−0.00155 (11)	0.9321 (4)	0.12181 (9)	0.0682 (5)	
C13	0.00381 (13)	1.1138 (4)	0.17359 (11)	0.0755 (5)	
H13	−0.0451	1.2186	0.1755	0.091*	
C14	0.08339 (13)	1.1327 (4)	0.22134 (10)	0.0722 (5)	
H14	0.0895	1.2520	0.2570	0.087*	
C15	0.15550 (12)	0.9762 (4)	0.21746 (8)	0.0611 (4)	
H15	0.2104	0.9875	0.2499	0.073*	
C16	−0.08995 (15)	0.7152 (6)	0.02254 (13)	0.1148 (9)	
H16A	−0.0495	0.7665	−0.0005	0.172*	
H16B	−0.1497	0.7207	−0.0068	0.172*	
H16C	−0.0766	0.5245	0.0400	0.172*	

### Atomic displacement parameters (Å^2^)

**Table:**

	*U*^11^	*U*^22^	*U*^33^	*U*^12^	*U*^13^	*U*^23^
O1	0.0609 (7)	0.0742 (7)	0.0434 (6)	−0.0007 (6)	0.0123 (5)	−0.0163 (5)
O2	0.0550 (8)	0.1215 (13)	0.1194 (12)	0.0237 (8)	0.0108 (8)	−0.0180 (11)
N1	0.0491 (7)	0.0441 (6)	0.0367 (6)	0.0006 (5)	0.0130 (5)	0.0019 (5)
N2	0.0561 (7)	0.0435 (6)	0.0385 (6)	0.0048 (6)	0.0166 (5)	0.0020 (5)
N3	0.0564 (8)	0.0524 (7)	0.0433 (7)	0.0002 (6)	0.0142 (6)	0.0009 (6)
N4	0.0473 (7)	0.0551 (8)	0.0505 (7)	0.0009 (6)	0.0129 (6)	−0.0120 (6)
N5	0.0477 (7)	0.0609 (8)	0.0643 (8)	0.0031 (6)	0.0175 (6)	−0.0004 (7)
C1	0.0482 (8)	0.0594 (9)	0.0465 (8)	0.0102 (7)	0.0139 (7)	0.0080 (7)
C2	0.0463 (8)	0.0567 (9)	0.0374 (7)	0.0026 (7)	0.0073 (6)	0.0039 (7)
C3	0.0462 (8)	0.0439 (7)	0.0312 (6)	−0.0011 (6)	0.0109 (6)	0.0050 (6)
C4	0.0765 (11)	0.0416 (8)	0.0490 (8)	0.0004 (8)	0.0240 (8)	−0.0025 (7)
C5	0.0505 (8)	0.0399 (7)	0.0428 (7)	0.0091 (6)	0.0175 (6)	−0.0028 (6)
C6	0.0583 (9)	0.0605 (10)	0.0550 (9)	0.0030 (8)	0.0235 (8)	0.0070 (8)
C7	0.0692 (11)	0.0741 (11)	0.0438 (8)	0.0168 (9)	0.0215 (8)	0.0106 (8)
C8	0.0682 (11)	0.0806 (12)	0.0412 (8)	0.0107 (10)	0.0050 (8)	−0.0063 (8)
C9	0.0570 (9)	0.0597 (10)	0.0525 (9)	−0.0012 (8)	0.0138 (7)	−0.0064 (8)
C10	0.0492 (8)	0.0478 (8)	0.0360 (7)	−0.0035 (7)	0.0141 (6)	0.0024 (6)
C11	0.0493 (8)	0.0467 (8)	0.0545 (9)	−0.0021 (7)	0.0222 (7)	0.0018 (7)
C12	0.0516 (10)	0.0708 (11)	0.0842 (13)	0.0081 (9)	0.0245 (9)	0.0051 (10)
C13	0.0673 (12)	0.0695 (12)	0.1041 (15)	0.0109 (10)	0.0478 (12)	0.0003 (11)
C14	0.0789 (13)	0.0668 (11)	0.0847 (13)	−0.0001 (10)	0.0455 (11)	−0.0141 (10)
C15	0.0621 (10)	0.0615 (10)	0.0648 (10)	−0.0035 (8)	0.0274 (8)	−0.0103 (8)
C16	0.0657 (13)	0.134 (2)	0.1161 (19)	0.0129 (14)	−0.0116 (13)	−0.0294 (18)

### Geometric parameters (Å, º)

**Table:**

O1—C10	1.2147 (16)	C4—H4A	0.9700
O2—C12	1.349 (2)	C4—H4B	0.9700
O2—C16	1.433 (3)	C5—C9	1.372 (2)
N1—C3	1.3354 (17)	C6—C7	1.367 (2)
N1—N2	1.3404 (16)	C6—H6	0.9300
N2—C1	1.3421 (18)	C7—C8	1.364 (3)
N2—C4	1.4476 (18)	C7—H7	0.9300
N3—C5	1.3271 (18)	C8—C9	1.378 (2)
N3—C6	1.3321 (19)	C8—H8	0.9300
N4—C10	1.3593 (18)	C9—H9	0.9300
N4—C11	1.3920 (19)	C11—C15	1.372 (2)
N4—H4N	0.859 (9)	C12—C13	1.385 (3)
N5—C12	1.317 (2)	C13—C14	1.357 (3)
N5—C11	1.338 (2)	C13—H13	0.9300
C1—C2	1.355 (2)	C14—C15	1.382 (2)
C1—H1	0.9300	C14—H14	0.9300
C2—C3	1.3893 (19)	C15—H15	0.9300
C2—H2	0.9300	C16—H16A	0.9600
C3—C10	1.477 (2)	C16—H16B	0.9600
C4—C5	1.508 (2)	C16—H16C	0.9600
			
C12—O2—C16	118.00 (16)	C8—C7—H7	121.0
C3—N1—N2	104.11 (11)	C6—C7—H7	121.0
N1—N2—C1	112.18 (12)	C7—C8—C9	119.21 (15)
N1—N2—C4	119.62 (12)	C7—C8—H8	120.4
C1—N2—C4	128.10 (13)	C9—C8—H8	120.4
C5—N3—C6	117.26 (13)	C5—C9—C8	118.76 (16)
C10—N4—C11	129.51 (13)	C5—C9—H9	120.6
C10—N4—H4N	114.6 (11)	C8—C9—H9	120.6
C11—N4—H4N	115.8 (11)	O1—C10—N4	124.52 (14)
C12—N5—C11	117.15 (15)	O1—C10—C3	122.02 (13)
N2—C1—C2	107.38 (13)	N4—C10—C3	113.46 (12)
N2—C1—H1	126.3	N5—C11—C15	123.41 (15)
C2—C1—H1	126.3	N5—C11—N4	112.27 (13)
C1—C2—C3	104.75 (13)	C15—C11—N4	124.32 (15)
C1—C2—H2	127.6	N5—C12—O2	119.06 (18)
C3—C2—H2	127.6	N5—C12—C13	124.14 (17)
N1—C3—C2	111.57 (13)	O2—C12—C13	116.80 (17)
N1—C3—C10	120.24 (12)	C14—C13—C12	117.31 (17)
C2—C3—C10	128.18 (13)	C14—C13—H13	121.3
N2—C4—C5	113.65 (12)	C12—C13—H13	121.3
N2—C4—H4A	108.8	C13—C14—C15	120.52 (17)
C5—C4—H4A	108.8	C13—C14—H14	119.7
N2—C4—H4B	108.8	C15—C14—H14	119.7
C5—C4—H4B	108.8	C11—C15—C14	117.47 (17)
H4A—C4—H4B	107.7	C11—C15—H15	121.3
N3—C5—C9	122.73 (14)	C14—C15—H15	121.3
N3—C5—C4	116.68 (13)	O2—C16—H16A	109.5
C9—C5—C4	120.50 (14)	O2—C16—H16B	109.5
N3—C6—C7	123.98 (16)	H16A—C16—H16B	109.5
N3—C6—H6	118.0	O2—C16—H16C	109.5
C7—C6—H6	118.0	H16A—C16—H16C	109.5
C8—C7—C6	118.04 (15)	H16B—C16—H16C	109.5
			
C3—N1—N2—C1	−0.62 (14)	C11—N4—C10—O1	0.4 (2)
C3—N1—N2—C4	−177.19 (11)	C11—N4—C10—C3	−179.08 (14)
N1—N2—C1—C2	0.51 (16)	N1—C3—C10—O1	176.69 (13)
C4—N2—C1—C2	176.71 (13)	C2—C3—C10—O1	−2.7 (2)
N2—C1—C2—C3	−0.17 (16)	N1—C3—C10—N4	−3.79 (18)
N2—N1—C3—C2	0.50 (14)	C2—C3—C10—N4	176.79 (14)
N2—N1—C3—C10	−179.01 (11)	C12—N5—C11—C15	−0.3 (2)
C1—C2—C3—N1	−0.21 (16)	C12—N5—C11—N4	179.54 (14)
C1—C2—C3—C10	179.25 (13)	C10—N4—C11—N5	175.29 (14)
N1—N2—C4—C5	83.78 (16)	C10—N4—C11—C15	−4.9 (3)
C1—N2—C4—C5	−92.17 (18)	C11—N5—C12—O2	−179.95 (16)
C6—N3—C5—C9	−0.7 (2)	C11—N5—C12—C13	0.5 (3)
C6—N3—C5—C4	175.81 (13)	C16—O2—C12—N5	4.2 (3)
N2—C4—C5—N3	37.45 (19)	C16—O2—C12—C13	−176.20 (19)
N2—C4—C5—C9	−145.99 (14)	N5—C12—C13—C14	−0.5 (3)
C5—N3—C6—C7	−1.0 (2)	O2—C12—C13—C14	179.96 (18)
N3—C6—C7—C8	1.5 (3)	C12—C13—C14—C15	0.2 (3)
C6—C7—C8—C9	−0.4 (3)	N5—C11—C15—C14	0.1 (2)
N3—C5—C9—C8	1.7 (2)	N4—C11—C15—C14	−179.76 (15)
C4—C5—C9—C8	−174.61 (14)	C13—C14—C15—C11	0.0 (3)
C7—C8—C9—C5	−1.2 (2)		

### Hydrogen-bond geometry (Å, º)

*Cg*1 is the centroid of the N1/N2/C1–C3 ring.

**Table:**

*D*—H···*A*	*D*—H	H···*A*	*D*···*A*	*D*—H···*A*
N4—H4*N*···N1	0.86 (1)	2.24 (2)	2.6939 (18)	113 (1)
C15—H15···O1	0.93	2.33	2.909 (2)	120
C10—O1···*Cg*1^i^	1.22 (1)	3.42 (1)	3.5486 (16)	86 (1)

Symmetry code: (i) *x*, *y*+1, *z*.
